# How to Improve the Implementation of Academic Clinical Pediatric Trials Involving Drug Therapy? A Qualitative Study of Multiple Stakeholders

**DOI:** 10.1371/journal.pone.0064516

**Published:** 2013-05-28

**Authors:** Delphine Girard, Olivier Bourdon, Hendy Abdoul, Sonia Prot-Labarthe, Françoise Brion, Annick Tibi, Corinne Alberti

**Affiliations:** 1 Université Paris Diderot, Sorbonne Paris Cité, Paris, France; 2 AP-HP, Hôpital Robert Debré, Unité d’Epidémiologie Clinique, Paris, France; 3 Inserm, CIE5, Paris, France; 4 Université Paris Descartes, Sorbonne Paris Cité, Paris, France; 5 AP-HP, Hôpital Robert Debré, Pharmacie à Usage Intérieur, Paris, France; 6 AP-HP, Agence Générale des Equipements et Produits de Santé, Unité d’Essais Cliniques, Paris, France; Nottingham University, United Kingdom

## Abstract

**Objective:**

The need for encouraging pediatric drug research is widely recognized. However, hospital-based clinical trials of drug treatments are extremely time-consuming, and delays in trial implementation are common. The objective of this qualitative study was to collect information on the perceptions and experience of health professionals involved in hospital-based pediatric drug trials.

**Methods:**

Two independent researchers conducted in-depth semi-structured interviews with principal investigators (n = 17), pharmacists (n = 7), sponsor representatives (n = 4), and drug regulatory agency representatives (n = 3) who participated in institutionally sponsored clinical trials of experimental drugs in pediatric patients between 2002 and 2008.

**Results:**

Dissatisfaction was reported by 67% (16/24) of principal investigators and pharmacists: all 7 pharmacists felt they were involved too late in the trial implementation process, whereas 11 (65%) principal investigators complained of an excessive regulatory burden and felt they were insufficiently involved in the basic research questions. Both groups perceived clinical trial implementation as burdensome and time-consuming. The sponsor and regulatory agency representatives reported a number of difficulties but were not dissatisfied.

**Conclusions:**

The heavy burden related to regulatory requirements, and suboptimal communication across disciplines involved, seem to be the main reasons for the major delays in pediatric drug trial implementation. The pharmaceutical aspects are intrinsically tied to trial methodology and implementation and must therefore be examined, in particular by involving Clinical Research Pharmacists at early stages of study conception.

## Introduction

In everyday practice, drug therapy in infants and children is often guided by personal experience and good intentions rather than by evidence from clinical trials. About 70% of drugs prescribed in children and up to 90% in neonates are unlicensed or used off-label (Appendix S1) [1]–[5]. Several reasons explain why clinical studies are performed less often in pediatric age groups than in adults. The specific ethical, methodological, and technical obstacles to pediatric trials are well recognized, as is the lack of financial rewards for the pharmaceutical industry [3], [6]–[10]. To address the paucity of pediatric research and to encourage investment by pharmaceutical companies, the United States and Europe have enacted new legislation about efficacy and safety drug trials in children [11]–[18]. In particular, the European Pediatric Regulation requires applications for marketing authorizations to be accompanied by either a product-specific waiver or a pediatric investigation plan, to be approved by the European Medicines Agency (EMA). In return, the patent is protected for an additional 6-month period. For older medicines not covered by a patent, a pediatric investigation plan can be devised in order to obtain a pediatric-use marketing authorization, which is associated with a patent that is protected for 10 years [19]. This regulation appears to have addressed the availability of medicines with age-appropriate information [20]. However, the impact on the number of clinical trials performed remains modest. Furthermore, studies done in the USA showed that drugs frequently used in pediatric patients were underrepresented among drugs qualifying for pediatric exclusivity [20]–[23].

Therefore, in addition to industry-based research, hospital-based investigations of pediatric drug therapy are needed [23]. However, the implementation of hospital-based clinical trials of drug treatments is extremely time-consuming. The resulting delays may be deleterious for the patients and may affect the competitiveness and attractiveness of clinical research in France [3], [24], [25].

The objective of this qualitative study was to identify obstacles to clinical trial implementation by collecting information on the perceptions and experience of health professionals involved in pediatric hospital-based research.

## Methods

### Ethics Statement

This study did not involve patients and did not require written consent. However, each selected participant was sent an information sheet explaining the study objectives. An e-mail was sent 3 days later to request consent to participation and to make an appointment. If no answer was received, a reminder was sent every 3 weeks, up to a maximum of three times. Anonymity and confidentiality of the interviews were guaranteed to all volunteers. At the beginning of each interview, the research objectives and confidentiality of study participation were reviewed with the participant, who was then asked to give oral consent and to allow audio recording of the interview. The Institutional Review Board of the Paris North Hospitals, Paris 7 University, Paris Public Hospital Network (AP-HP) approved the study protocol, including the information sheet and oral consent procedure (N° 12-049).

### French Hospital-based Biomedical Research

The French hospital-based research system has four main components.

- **The DIRCs** (*Délégation Interrégionale à la Recherche Clinique*) sponsor hospital-based clinical research and manage administrative, legal, and financial issues. Every French university hospital is affiliated with one of the seven DIRCs. The DIRC for the Ile-de-France region area includes a vast division, the AP-HP (*Assistance Publique-Hôpitaux de Paris*), located in the Paris conurbation. This study focused on AP-HP-sponsored research.

- **The methodological units** are under the authority of the DIRCs. Their main roles are to manage methodological and biostatistical issues, contribute to manage legal issues, and monitor the studies.

- **The ANSM** (*Agence Nationale de Sécurité du Médicament et des Produits de Santé,* previously named AFSSAPS) is a national institution under the authority of the Ministry of Health. It is the delegated health authority; the competent authority for French biomedical research involving drug therapy; and the French registration authority for all products and techniques used in biomedical research.

- **Central and/or local hospital pharmacies** are in charge of the pharmaceutical process. Central hospital pharmacies are specific entities, of which each is affiliated with a public hospital network.

### Selection of Participants

All principal investigators, pharmacists, Ile-de-France DIRC representatives (i.e., sponsor representatives), and ANSM representatives who had participated in a clinical AP-HP-sponsored trial involving a pediatric population and experimental drugs between January 1, 2002, and December 31, 2008 were eligible. These criteria were met by 38 principal investigators (all medical doctors), 11 pharmacists from hospital pharmacies, 1 pharmacist from the central hospital pharmacy, 19 sponsor representatives, and 3 experts working in the ANSM pediatrics unit. Samples of principal investigators and sponsor representatives were taken at random. For the principal investigators, the random selection procedure was stratified on the hospital in which they worked.

### Interviews

Four interview guides were designed for the semi-structured interviews of the principal investigators, pharmacists, sponsor representatives, and ANSM representatives, respectively. Each guide focused on the same four dimensions of trial implementation: establishment of collaborations among the various health professionals involved, pharmaceutical issues, financial aspects, and education for clinical research (Table 1). Before the study, all authors worked together to define these four dimensions based on an analysis of the medical and sociological literature.

**Table 1:** Topics covered in the interviews.

**Establishment of the collaboration** between the different health professionals involved
Timing of the establishment of the collaboration
Perception of the collaboration
**Pharmaceutical issues**
Procurement of the experimental drugs
Dosage form design and development
**Budgetary aspects**
Evaluation of costs
Match between the funds obtained and the funds needed
**Professional training for clinical research**
Investigator training before involvement

The interviews used open questions to allow the participants to discuss aspects they felt were important. All interviews occurred at the interviewee’s workplace, face-to-face for the principal investigators and the pharmacists and in focus groups for the sponsor and ANSM representatives. One interviewer (DG), trained by a sociologist (CP), conducted all interviews. Neutrality was ensured by the fact that DG, a resident pharmacist, had never been involved in the implementation of a clinical trial.

All interviews were audio-recorded and transcribed verbatim anonymously by an independent person (CUO) who was not otherwise involved in the study. One recording was of insufficient quality to allow transcription; however, the written notes taken during this interview allowed a valid analysis. Biographical information for each participant was collected at the beginning of each interview. Interviews were continued until the saturation point [26], [27] was reached, *i.e.*, until no new information could be gathered by an additional interview. The saturation point in qualitative studies is usually reached between 20 and 30 interviews [27]–[29].

### Analysis

The transcribed interviews were analyzed and coded by DG and HA, who used both case-oriented and variable-oriented methods [30]. Each interview was parsed by theme, and recurring themes were identified inferentially. Similarities and differences for variables in each theme were listed. DG, HA, and CA discussed the development of the themes and variables and validated the process. In addition, cross-validation of the thematic analysis was performed simultaneously by DG using the text analysis software Tropes® (Semantic Knowledge, France) [31]. The results were compared and discussed with all authors.

Three topics pertaining to the implementation of pediatric trials involving drug therapy were identified: global perception of implementation, problems with implementation, and solutions. The results are reported according to RATS qualitative research review guidelines [32].

## Results

### Characteristics of Participants and Description of Interviews

All 12 pharmacists and all 3 ANSM representatives were invited to participate, as were 26 of the 38 eligible principal investigators and 4 of the 19 sponsor representatives. Nine principal investigators and 3 pharmacists did not reply; in addition, 2 pharmacists refused to participate (mainly because they felt insufficiently experienced in pediatric research). Therefore, the study was done in 17 principal investigators (including 12 pediatricians), 7 pharmacists, 4 sponsor representatives, and 3 ANSM representatives. Table 2 shows the participant characteristics. Age was 40 to 50 years in 52% of cases, and 58% of participants were women. Of the 17 principal investigators, 11 (65%) had experience with a single pediatric drug trial. Nonparticipants had a median age of 51 years (range, 48-57 years) and 71% of them were women. Median interview duration was 30 minutes. In general, the participants were grateful for the opportunity to express their concerns. Appendix S2 shows characteristics of participants cited in the article.

**Table 2:** Characteristics of participants.

	Principal investigators (n = 17)	Pharmacists (n = 7)	Sponsor representatives (n = 4)	ANSM representatives (n = 3)	Total (n = 31)
**Age,** n(%), years					
[30]–[40]	–	28.6(2)	25.0(1)	66.7(2)	5(16.1)
[40]–[50]	52.9(9)	57.1(4)	50.0(2)	33.3(1)	16(51.6)
[50–60]	47.1(8)	14.3(1)	25.0(1)	–	10(32.3)
**Sex,** n(%)					
Male	8 (47)	3 (43)	2 (50)	–	13 (42)
Female	9 (53)	4 (57)	2 (50)	3(100)	18 (58)
**Workplace,** n(%)					
Pediatric hospital	10 (59)	3 (43)	–	–	13 (42)
General hospital	7 (41)	3 (43)	–	–	10 (32)
Central Pharmacy	–	1 (14)	–	–	1 (3)
DIRC	–	–	4 (100)	–	4 (13)
ANSM	–	–	–	3(100)	3(10)
**Specialty,** n (%)					
General pediatrics	2 (12)	–	–	–	2 (12)
Pediatric neurology	2 (12)	–	–	–	2 (12)
Pediatric pulmonology	2 (12)	–	–	–	2 (12)
Pediatric hematology	2 (12)	–	–	–	2 (12)
Pediatric nephrology	2 (12)	–	–	–	2 (12)
Neonatology	2 (12)	–	–	–	2 (12)
Pediatric pharmacology	1 (6)	–	–	–	1 (6)
Pediatric orthopedic surgery	1 (6)	–	–	–	1 (6)
Pediatric emergencies	1 (6)	–	–	–	1 (6)
Physiology	1 (6)	–	–	–	1 (6)
Metabolism and diabetes	1 (6)	–	–	–	1 (6)
**First clinical trial,** n (%)					
Yes	11 (65)	–	–	–	11 (39)
No	6 (35)	7 (100)	4 (100)	3(100)	17 (61)

**Abbreviations** ANSM, *Agence Nationale de Sécurité du Médicament et des Produits de Santé;* DIRC, *Délégation Interrégionale à la Recherche Clinique*.

### Global Perception of Trial Implementation

Table 3 shows the results for each of the three topics. Among the principal investigators and pharmacists, 67% (16/24) were dissatisfied. The reasons differed between principal investigators and pharmacists: all 7 pharmacists felt they were involved too late in the trial implementation process, and 65% (11/17) of principal investigators complained that the regulatory process was excessively burdensome and that they were insufficiently involved in the basic research questions. Both groups perceived trial implementation as onerous and time-consuming.

**Table 3:** Synthesis of the results.

Global perception of trial implementation
Pharmacists
- dissatisfaction
- late involvement
- secondary importance of pharmaceutical issues
Principal investigators
- dissatisfaction
- time-consuming
- a struggle
- disheartening
Sponsor representatives
- implementation of pediatric trials globally more complicated than for trials in adults
- principal investigators discouraged by the complexity of implementation
ANSM representatives
- no specific perception of the implementation of hospital-based studies compared to pharmaceutical company-based studies
**Key factors affecting the perception of trial implementation**
Pharmacists
- study schedule defined without involving the pharmacists
Principal investigators
- excessive number of parties involved
- regulatory burden
- complexity of the pharmaceutical process
Pharmacists and sponsor representatives
- acquisition of drug therapy in an appropriate formulation
- extemporaneous drug preparation
- authorization by regulatory agency
- inadequate funds
ANSM representatives
- no specific perception of the implementation of hospital-based studies
**Solutions**
Pharmacists and principal investigators
- to establish relationships with the pharmaceutical companies
Principal investigators
- to involve the pharmacists earlier
- to train investigators
- to simplify the process : “a single window approach”
Pharmacists and sponsor representatives
- to perform pharmaceutical feasibility assessments during study protocol development
ANSM representatives
- to rely more heavily on EMA and ANSM support systems during trial implementation
- to consider the specific features of pediatric trials early in the process
All stakeholders
- to improve communication among the disciplines involved

**Abbreviations:** AP-HP, *Assistance Publique-Hôpitaux de Paris;* EMA, *European Medicines Agency*; ANSM, *Agence Nationale de Sécurité du Médicament et des Produits de Santé*.

The **pharmacists** often described participation in a clinical trial as “working in distress” due to their late involvement in the process. They felt that the pharmaceutical issues were often considered of secondary importance.

*“That is how study protocols are selected. If the scientific question is interesting and relevant, the protocol is considered worthy of attention, but the pharmaceutical issues are viewed as unimportant and always surmountable. But this is how the studies become utterly exhausting.” (Int 19– pharmacist).*

Of the 17 **principal investigators**, 12 (71%) felt that implementing a clinical drug trial was so time-consuming, complex, and discouraging that investigators might be unwilling to participate in further trials.

*“My first thought was that in the future I will only participate in trials that do not deal with medications. It is not manageable for a clinician working alone. The time and effort needed are enormous, and it is just not worth it under the present conditions.” (Int 4– principal investigator).*

In contrast, the sponsor and ANSM representatives described a number of difficulties but were not dissatisfied.

**Sponsor representatives** felt that pediatric trials were globally more complicated to implement than trials in adults.

*“The pediatric population is a high-risk population, which complicates everything.” (Int 28– sponsor representative).*

They felt that this complexity discouraged principal investigators.

*“There are few pediatric drug trials because investigators don’t want to get involved in them any more. They know that these trials involve months and months of struggling.” (Int 26– sponsor representative).*

The **ANSM representatives** explained that their agency conducts a standardized assessment that is the same for all biomedical studies, regardless of the sponsor. As a result, they had no specific perceptions of the implementation of hospital-based studies.

### Pharmacists’ Perceptions of Trial Implementation

The pharmacists identified a number of challenges related to the implementation of pediatric clinical trials. The first point concerned drug acquisition. Contract negotiations with the pharmaceutical companies were identified as a key difficulty in clinical trial implementation. The divergent needs of the pharmaceutical industry and academic researchers often gave rise to contentious issues, which could result in long delays.

*“We negotiated with the laboratory for a year about getting the drug and the placebo. We finally never got it, because meanwhile they obtained a license to use the drug for another indication in adults. They took us for a ride.” (Int 23– pharmacist).*

The second point concerned the suitability of available galenic formulations (Appendix S1) to age and route of administration. If no suitable galenic formulation was available, extemporaneous formulations had to be prepared. These preparations were produced by the hospital pharmacy or by a subcontractor. In both situations, they delayed study initiation.

*“It was very complicated to get the drug formulation we needed, because at first the company promised to provide us with the drug in powder form, and we set up all the procedures for the powder. But then they changed their mind and decided to provide tablets, which meant we had to crush them before use. Therefore, we had to purchase special equipment for crushing the tablets and we had to perform additional analyses to check that the crushed tablets were stable and physicochemically suitable for our needs.” (Int 20– pharmacist).*

In addition, extemporaneous drug preparations (Appendix S1) used in clinical trials must be approved by regulatory agencies, which can refuse the use of an extemporaneous preparation of a drug that is widely prescribed in everyday practice. Finally, the study schedule was often established without involving the pharmacists. The result was sometimes a mismatch between drug production constraints and patient visit time-points. Overall, because pharmaceutical needs were not anticipated, pharmacists had to face unexpected costs.

*“The amount of tablets to be produced was too small, so the pharmaceutical company did not want to produce them. So finally the hospital pharmacy had to produce the drug, although no funds had been set aside for that purpose.” (Int 24– pharmacist).*

### Principal Investigators’ Perceptions of Trial Implementation

The principal investigators felt that too many parties were involved during trial implementation and that coordination among these parties was poor. The perceived result was inadequate communication and loss of information.

*“It was a lot of work to finally manage to get everybody together, because the number of persons involved is just crazy. I really think that this jeopardizes the good conduct of a study.” (Int 12– investigator).*

The principal investigators felt that the burden created by the regulatory procedures was excessive. Among them, 60% reported spending more time on administrative and regulatory issues than on scientific ones.

*“This kind of work is not what we studied medicine for: too much administration, too much clerical work. Instead, we should spend most of our time on conceiving the project and writing the medical part of the study protocol.” (Int 13– investigator).*

They also complained of lack of flexibility of the regulatory agencies. In their opinion, this lack of flexibility was the result of undue fear of liability.

*“Is all this really for the safety of the patients or is it to protect the regulatory agencies?” (Int 4– investigator).*

*“A major problem in clinical research is that the same strict regulatory rules apply for every protocol, which is not always justified.” (Int 11– investigator).*

The principal investigators felt that the regulatory complexities caused inertia and made a major contribution to the substantial delays in implementing trials involving drug therapy. This slowness was unacceptable to the medical doctors, for two main reasons. First, they pointed out that making patients wait for treatment because of “administrative factors” was ethically unacceptable.

*“During this administrative process, I have seen children who were waiting to participate in the protocol and whose clinical condition worsened. In the end, they could not participate, because they had lost the ability to walk, which was an exclusion criterion.” (Int 5– investigator).*

Second, the investigators described delays as potentially deleterious for the relevance of the scientific question of the trial and as adversely affecting the competitiveness of French clinical research.

*“I am not sure whether we will be able to include the number of patients needed. At that time, the question was of paramount importance, but now, 3 years later, of course it is not as interesting any more.” (Int 4– investigator).*

*“I had to struggle for more than a year to get everything set up, so we were the last country to include patients. All the other European countries started their inclusions before us.” (Int 13– investigator).*

When asked about pharmaceutical issues, 12 (71%) principal investigators reported difficulties, describing a lengthy process and a need to overcome many obstacles. They were not aware of the details and stated that they usually left these issues to the pharmacists.

Despite the major delays in trial implementation related to pharmaceutical issues, the principal investigators acknowledged the pharmacists’ commitment.

*“It was very complicated, but there was a lot of goodwill on the part of the pharmacists. It took us 1 year to organize the pharmaceutical processes. The pharmaceutical company did not want to get involved so we had to find a way to obtain and prepare the drug. The pharmacists took care of all these issues, and I believe it was very difficult and time-consuming for them. ” (Int 11– investigator).*

Among the principal investigators, 7 (41%) felt that earlier involvement of the pharmacists in trial implementation was important.

*“We contacted them early and asked for their opinion about the feasibility of the pharmaceutical processes. They were really helpful. They allowed us to anticipate many problems and to find solutions, so that the process moved forward smoothly.” (Int 8– investigator).*

### Sponsor Representatives’ Perceptions of Trial Implementation

The 4 sponsor representatives pointed out that pediatric trials had specific features and emphasized the greater stringency of regulations for pediatric populations compared to adult populations. Moreover, 3 of the 4 sponsor representatives identified a number of specific drug-related issues in pediatric populations. Similar to the pharmacists, they pointed out that acquiring the study drugs was the main problem. They also highlighted the difficulties in establishing contracts with the pharmaceutical companies and the lack of available pediatric formulations.

*“Pharmaceutical companies are not interested in pediatric trials, so contract negotiations can be very complicated. They don’t want to get their drugs involved in high-risk trials, especially since there is no financial reward.” (Int 26– sponsor’s representative).*

These specific features added costs, which were often not anticipated. Moreover, when they were anticipated, adequate funding was often not granted.

*“If the industry doesn’t want to help you, then you have to develop a galenic formulation for the trial yourself. Why not … But how can you do this if you don’t have enough money? And if you have to prepare a placebo – well, that’s even much worse!” (Int 27– sponsor’s representative).*

### ANSM Representatives’ Perceptions of Trial Implementation

The ANSM representatives highlighted the specific pharmaceutical issues in pediatric drug development and the importance of considering these issues as early as possible. The ANSM has issued several guidelines to help with these issues. In addition, in 2008 the ANSM opened a support unit to help study designers work through the specific pharmaceutical issues encountered in the pediatric population. The ANSM representatives felt that these tools were insufficiently used.

### Suggestions for Improving the Implementation of Pediatric Clinical Trials Involving Drug Therapy

The pharmacists said they wanted to be involved earlier in the implementation process, so they could anticipate pharmaceutical issues. As the sponsor’s representatives, they felt that documentation of pharmaceutical feasibility should be a prerequisite to grant allocation. This measure would ensure adequate funding for the pharmacy and would avoid grant allocation to projects having insurmountable pharmaceutical obstacles.

*“During the evaluation process of a clinical study, the experts concentrate on the aim and methods, but nobody cares if the study is feasible from the pharmacy’s point of view.” (Int 19– pharmacist).*

*“Medical doctors think more about the publication of their results than about pharmaceutical feasibility when they design the protocol; for example, they always want to perform double-blind placebo-controlled clinical trials without giving enough thought to placebo procurement, cost, feasibility of double blinding …” (Int 26– Sponsor’s representative).*

A first step toward improving trial implementation would be to increase awareness among medical doctors of potential pharmaceutical difficulties.

*“Clinicians do not understand the pharmaceutical constraints. They call you 3 weeks before they want to submit the project and ask ‘Look, we are going to submit this, what do 50 tablets cost?’” (Int 19– pharmacist).*

The ANSM representatives felt that education and communication about the European Medicines Agency (EMA) and ANSM support systems were needed.

*“It is not normal that the first pharmaceutical assessment occurs after the study has acquired funding, although support can be obtained from the ANSM and EMA.” (Int 29– ANSM representative).*

Of the 17 principal investigators, 6 (35%) said they failed to accurately predict the time needed for study implementation, because they were not aware of the different steps. They indicated a desire to receive education on this topic.

*“I think it is important to become familiar with research issues during one’s residency at the latest. Future pediatricians must be aware of some of the aspects of clinical research.” (Int 14– principal investigator).*

Establishing relationships with the pharmaceutical industry, particularly with start-up companies, was viewed favorably by 57% (n = 4) of the pharmacists and 47% (n = 8) of the principal investigators.

*“Clinical trials require both an appropriate infrastructure and manpower. Special pharmacies having such resources could be set up for pediatric studies. We would then be able to offer specialized services to the pharmaceutical industry, such as taking over small-scale production, which costs them a fortune. The money thus earned might be sufficient to fund the special pharmacy.” (Int 19– pharmacist).*

## Discussion

### Main Results

This study documented major differences in perceptions of the implementation of institutional pediatric clinical trials across professional groups. Principal investigators emphasized the time-consuming nature of trial implementation, which they ascribed in large part to the enormous amount of paperwork needed to comply with regulations. Pharmacists, in contrast, complained chiefly of being involved too late in the trial implementation process. Interestingly, the problems experienced by principal investigators were not specific to pediatric trials, whereas the pharmacists reported difficulties in obtaining drugs and formulations for pediatric patients. Overall, principal investigators seemed to lack awareness of the specific pharmaceutical requirements for pediatric clinical trials, especially during their first such trial.

Sponsor representatives felt that principal investigators were discouraged about pediatric clinical trials. On the one hand, they pointed out the greater stringency of regulations for trials in pediatric populations and, on the other, they felt that failure to anticipate pharmaceutical problems was common. They emphasized the need to educate principal investigators about the specific pharmaceutical issues raised by pediatric trials.

The ANSM is aware of the difficulties raised by implementing pediatric clinical trials and has issued several support tools to help both industrials and academics in planning their research projects.

### Strengths and Weaknesses of the Study (Internal Validity)

We studied perceptions and experiences of several types of stakeholders regarding the implementation of institutionally sponsored pediatric clinical trials of experimental drugs. Clinical research nurses are assuming a growing role in therapeutic trials in many countries [33]–[35]. However, there are few clinical research nurses in France, and their role remains ill-defined. Clinical research nurses were not identified in any of the protocols for pediatric trials of experimental drugs sponsored by the AP-HP between 2002 and 2008.

We chose semi-structured interviews to explore participants’ opinions without inducing or guiding their answers. Results of qualitative studies should not be extrapolated to the general population. In keeping with qualitative study principles, our objective was not to obtain quantitative data or an exhaustive list of participants’ views but, instead, to identify different types of obstacles to the implementation of pediatric clinical trials involving drug therapy. To ensure reliability of our results, the interviews were analyzed by two researchers working independently of each other. In addition, the interviews were also evaluated using text analysis software.

### Strengths and Weaknesses of the Study Compared to Other Studies (External Validity)

We focused on AP-HP sponsored studies and therefore on a single geographic region (the Paris conurbation), which may limit the external validity of our findings. However, the AP-HP is the leading academic sponsor in France and the leading research center in Europe. Moreover, the results of our study highlight issues that are not specific of the AP-HP institution. Indeed, burdensome regulatory requirements [3], [25], [36], pharmaceutical issues [1], [37]–[39], and difficulties in establishing contracts with pharmaceutical companies [25] have been reported internationally.

Numerous publications address off-label and unlicensed drug use in pediatrics [1], [2], the continuing paucity of data on pediatric formulations [37], [40], and other difficulties in obtaining age-appropriate drug formulations for the pediatric population [38], [39], [41]. In contrast, the obstacles faced by pharmacists in the implementation of academic clinical trials are not mentioned in articles reporting academic pediatric clinical trials [3]. Thus, no information is given on difficulties in procuring drugs, contract negotiations with pharmaceutical companies, or timeline management [42]. Pharmacists must be encouraged to publish their data about these issues.

Many of the problems described by the principal investigators in our study have been discussed in the literature, including the large amount of administrative work [43] and the burden created by regulations [25], which cause some medical doctors to refuse participation in clinical trials [3]; the fact that clinical practice and clinical research are intrinsically different activities [44]–[46]; and the feeling reported by investigators of being overwhelmed at times [3], [24], [36], [47].

The need for a partnership between university hospitals and the pharmaceutical industry has also been discussed previously. Osuntokun emphasized the importance of developing a closer working relationship with the pharmaceutical industry to ensure improved pediatric drug labeling [3]. Pons highlighted the importance of a partnership between academic pediatricians, the government, and the pharmaceutical industry [14].

### Meaning of the Study Results and Implications for Policymakers

This study identifies the heavy regulatory burden, inadequate training, and poor communication among disciplines as the main reasons for the major delays in academic pediatric drug trial implementation.

The 2001 European directive led to significant improvements in patient safety, trial validity, and data reliability. However, the complexity of this directive and the variability in its interpretations across European Union countries have been recognized as contributors to the loss of European competitiveness in research over the past decade. In July 2012, the European Commission finalized a proposal for a Clinical Trial Regulation, which will replace the 2001 European directive [48]. The regulation will aim to facilitate the implementation of clinical trials and to reduce the regulatory burden via a risk-proportionate approach, to reduce implementation delays and costs. Thus, regulatory requirements and restrictions will be proportionate to the risk incurred by study patients (e.g., knowledge of the experimental drug, type of intervention). Journot et al. demonstrated the value of a risk-based monitoring strategy for academic clinical research studies [49].

Training programs for future investigators might be helpful, particularly if they emphasize the specific issues raised by pediatric drug trials. Current training delivered to investigators focuses on methodological issues. A stronger emphasis must be placed on pharmaceutical and regulatory difficulties. Investigators who are cognizant of those difficulties are more likely to anticipate them and therefore to devise early solutions.

Several of the pharmaceutical problems could be prevented by performing pharmaceutical feasibility assessments early in the process, *i.e.,* while designing the trials. Pharmaceutical expertise is particularly important for pediatric clinical trials, since these raise complex issues of drug acquisition and formulation. The difficulties vary in magnitude depending on the experimental drugs and pharmaceutical companies involved, number of groups and of patients, treatment duration and dosage, drug shelf life, and other factors. Pharmacists are well able to predict and to circumvent problems in pediatric clinical trial implementation and can therefore help to determine the time and funds needed. For all these reasons, in addition to the recently introduced “Clinical Research Pharmacist” concept [50], [51], we may need to encourage the development of “research pharmacists” trained in providing early pharmaceutical expertise and in making recommendations consistent with methodological constraints.

### Conclusions

The enormous regulatory burden and suboptimal communication across disciplines involved seem to be the main reasons for the major delays in academic pediatric drug trial implementation. Most of the pharmaceutical problems could be prevented by performing pharmaceutical feasibility assessments during the development of the study protocol. The pharmaceutical aspects are intrinsically tied to trial methodology and implementation and must therefore be examined particularly by involving research pharmacists at early stages of study conception.

## Supporting Information

Appendix S1 **Definitions.**
(DOC)

Appendix S2 **Characteristics of participants cited in the article.**
(DOC)
