# Supplementary material for: How to Improve the Implementation of Academic Clinical Pediatric Trials Involving Drug Therapy? A Qualitative Study of Multiple Stakeholders
Source: PLoS One. 2013 May 28;8(5):e64516. doi: 10.1371/journal.pone.0064516 (PMC3665797; doi:10.1371/journal.pone.0064516)
Supplement: Appendix S1 — Definitions. (DOC) [file pone.0064516.s001.doc]

Appendix S1. Definitions

| **Off label** |
| --- |
| The use of a licensed medication outside the specifications of its licensed use with regard to its indications, dosing, patient age group, route of administration, contraindications, etc. |
| **Unlicensed medication use** |
| The use of a medication that may only be used if licensed by the responsible government authority, but that has not been licensed. |
| **Pediatric drug formulation** |
| Dosage format ensuring the accurate, safe, effective, and high-adherence administration of a medication to children of various ages. |
| **Galenic formulation** |
| Galenic science deals with the principles of preparing and compounding medicines in order to optimize their absorption. The galenic formulation refers to the pharmaceutical dosage form of a medicine. |
| **Extemporaneous preparation** |
| Extemporaneous preparations are produced by pharmacists from various drug and chemical ingredients, using traditional compounding techniques to obtain suitable forms of medicines when no commercial form is available. |
