# Supplementary material for: How to Improve the Implementation of Academic Clinical Pediatric Trials Involving Drug Therapy? A Qualitative Study of Multiple Stakeholders
Source: PLoS One. 2013 May 28;8(5):e64516. doi: 10.1371/journal.pone.0064516 (PMC3665797; doi:10.1371/journal.pone.0064516)
Supplement: Appendix S2 — Characteristics of participants cited in the article. (DOC) [file pone.0064516.s002.doc]

Appendix S2. Characteristics of participants cited in the article

| **Participant #** | **Sex** | **Age (years)** | **Specialty** |
| --- | --- | --- | --- |
| **Principal Investigators** |  |  |  |
| Int 4 | Male | [50-60[ | Pediatric nephrology |
| Int 5 | Female | [40-50[ | Pediatric neurology |
| Int 8 | Male | [50-60[ | Pediatric emergencies |
| Int 11 | Male | [50-60[ | Pediatric neurology |
| Int 12 | Female | [40-50[ | Pediatric pulmonology |
| Int 13 | Male | [50-60[ | Pediatric hematology |
| Int 14 | Male | [50-60[ | Pediatric hematology |
| **Pharmacists** |  |  |  |
| Int 18 | Male | [30-40[ | - |
| Int 19 | Male | [40-50[ | - |
| Int 20 | Male | [30-40[ | - |
| Int 23 | Female | [40-50[ | - |
| Int 24 | Female | [40-50[ | - |
| **Sponsor representatives** |  |  |  |
| Int 26 | Male | [50-60[ | - |
| Int 27 | Female | [30-40[ | - |
| Int 28 | Female | [40-50[ | - |
| **ANSM representatives** |  |  |  |
| Int 29 | Female | [40-50[ | - |
